# Risk factors for late complications in pediatric penetrating brain injuries: a retrospective study

**DOI:** 10.3389/fneur.2026.1832612

**Published:** 2026-08-10

**Authors:** Tao Huang, Jingxi Du, Gaoyang Zhou, Zhen Wang, Wei Guo, Chen Yang, Shaochun Guo, Zhihong Li, Julei Wang

**Affiliations:** 1 Department of Neurosurgery, Tangdu Hospital of Air Force Medical University, Xi’an, China; 2 Department of Ultrasound medicine, Tangdu Hospital of Air Force Medical University, Xi’an, China; 3 Department of Medical Imaging, Hospital of Unit 96608, PLA, Hanzhong, China

**Keywords:** children, epilepsy, late complications, materials, pediatric brain injuries

## Abstract

**Objective:**

Pediatric penetrating brain injuries (PBIs) represent a significant public health challenge among children, contributing substantially to global morbidity and mortality. The long-term complications following such trauma can profoundly affect the psychological and physical development of affected children. This study aims to investigate the risk factors associated with late complications resulting from penetrating brain injuries in pediatric patients.

**Methods:**

This single-center retrospective cohort study included 30 children with PBIs. Among these, 19 cases involved penetrating brain injuries caused by metallic objects (such as rebar, nails, darts, and scissors), while the remaining 11 cases were due to wooden materials (such as branches and chopsticks). Clinical and laboratory data were collected and systematically analyzed. Both univariate and multivariate analyses were conducted on the variables.

**Results:**

In the univariate analysis, the severity of brain-penetrating injury (OR = 3.864, *p* = 0.038) and the depth of penetration (OR = 5.867, *p* = 0.001) are identified as significant risk factors for delayed complications. In the multivariate analysis, depth of penetration (OR 8.132, *p* = 0.012) was identified as an independent risk factor for the incidence of delayed complications. Statistical analysis using Fisher’s exact test revealed that the type of material (*p* = 0.003) and the entry point (*p* = 0.018) are significant factors associated with late epilepsy.

**Conclusion:**

Depth of penetration may be associated with late complications. The material of the injury and entry point are associated with the late epilepsy. However, due to the small sample size and limited control of confounding factors, this association is exploratory and requires confirmation through larger prospective or multicenter studies.

## Introduction

The most common causes of brain trauma in children are falls, while more serious craniocerebral injuries are associated with traffic accidents (1). Brain injury cases among children have increased significantly (2), resulting in an enormous socio-economic burden. Brain injuries are the main causes of death and disability, accounting for >50% of deaths in pediatric populations (3).

Penetrating brain injuries (PBIs) are usually caused by firearms in wartimes and by sharp instruments such as steel bars, daggers, chopsticks, pencils, and steel nails in non-wartimes. The unsupervised use of sharp objects is a major cause of penetrating craniocerebral injuries in pediatric patients. Patients with penetrating craniocerebral injuries are often in critical condition, often accompanied by hemorrhagic shock or brain injury and infection, with a high mortality rate. The involvement of functional areas in these injuries might result in obvious sequelae, resulting in difficulties in neurosurgical treatment. PBIs constitute approximately 0.4% of all head injuries and are typically the result of falls, motor vehicle collisions, and explosions (4).

PBI neurosurgical management is based on correcting the entry point to avoid cerebral spinal fluid (CSF) leakage (primary lesion) and treating secondary lesions caused by the penetrating object, such as brain parenchyma and vascular lesions (5). Thus, preoperative neuroradiologic assessment is vital for the correct neurosurgical approach.

Penetrating brain injuries can lead to significant damage to the central nervous system and are correlated with elevated mortality rates and prolonged disability. Survivors frequently face the risk of persistent neurological complications that considerably diminish their quality of life. This study seeks to investigate the risk factors associated with long-term complications in pediatric patients who have sustained penetrating brain injuries, specifically during the postoperative follow-up period. The objective is to furnish clinical evidence that may enhance prognostic outcomes for this population.

## Methods

### Patients

We conducted a retrospective, single-center study, which received approval from our institutional ethics committee for data collection. Written informed consent was obtained from the parents or guardians prior to data collection. We retrospectively analyzed a series of pediatric patients with confirmed TBI at Tangdu Hospital and reviewed published studies to gain a neurosurgical management approach to PBI. At our institution, 30 patients had confirmed PBI between 2013 and 2024. From January 2013 to December 2024, 200 pediatric patients presenting with TBI were admitted to the Tangdu Hospital Department of Neurosurgery. From those patients, we selected 30 patients presenting lesions caused by penetrating objects. Patients with objects penetrating the oral cavity were also assessed by a maxillofacial dentist. Data were retrospectively extracted from medical records, encompassing the following variables: gender, weight, entry point, Classification of Severity for Penetrating Brain Injury (PBI) Based on Admission Glasgow Coma Scale (GCS): Mild (GCS 13–15), Moderate (GCS 9–12), and Severe (GCS 3–8), discharge GCS score, readmission status, follow-up visit Karnofsky Performance Status (KPS) score, depth of penetration(the measurement was conducted using CT scans), and late complications (no postoperative complications were observed during the initial recovery period; however, new complications developed 3 months post-surgery). The inclusion criteria specified penetrating brain injuries (PBIs) in patients aged 18 years or younger. This study included patients undergoing surgical treatment for the first time at our center. Exclusion criteria included cases of comprehensive injury, coagulation dysfunction, and immediate postoperative complications such as epilepsy and cerebrospinal fluid leakage. Clarification of abscess case inclusion criteria (early 3-month cases excluded).

### The definition of late complications

The definition of late complications is articulated as follows: patients exhibited no postoperative complications during the initial recovery phase, yet new adverse events emerged 3 months postoperatively. The diagnosis of epilepsy was established based on the presence of typical clinical seizure manifestations in children, a comprehensive clinical evaluation by neurosurgeons, and the identification of epileptiform discharges on electroencephalography (EEG). The diagnosis of brain abscess primarily depended on the characteristic imaging findings observed in contrast-enhanced cranial MRI or CT scans. Cerebrospinal fluid leakage was confirmed through the presence of typical clinical symptoms in conjunction with high-resolution CT or MRI scans. The assessment of headaches was conducted by documenting patients’ self-reported pain location, character, severity, duration, and precipitating or relieving factors.

### Standardized perioperative antibiotic prophylaxis protocol

A standardized, institution-wide protocol for perioperative antibiotic prophylaxis was uniformly implemented for all patients, ensuring consistency in the selection of antibiotics, dosing regimens, and duration of administration.

### Classification of penetrating objects

Objects that penetrate through materials can be classified into metal types (such as steel bars, nails, scissors) and wood types (such as branches, chopsticks).

### Data evaluation

The IBM SPSS 21.0 (IBM Corp., NY, USA) was used for data analysis. Normal distribution of data was analyzed by Kolmogorov–Smirnov test and was expressed as the mean ± SD. Non-normally distributed data and ordinal data were expressed as median with inter-quartile range (IQR). Unpaired *t*-test was used for the comparisons of parameters with normal distribution between two groups and Mann–Whitney U test was used when in parameters without normal distribution. Categorical data was expressed as number (percentage) and compared by Chi-square χ^2^ test or Fisher’s exact test. A two-sided *p* value < 0.05 was regarded as statistically significant.

## Results

### Patient selection

Among the cohort of 30 patients, 20 individuals (66.7%) were male, while 10 individuals (33.3%) were female. The age range of the patients was 2 to 14 years, with a mean age of 8.0 years and a standard deviation of 3.3 years. The median follow-up duration was 22 months (IQR: 15–36). All patients underwent neuroradiological assessment and subsequently received neurosurgical intervention involving the removal of the foreign body via craniotomy. Notably, there were no fatalities reported. Significant differences in entry points were observed between the metal material group and the wooden material group (*p* < 0.001). There were no statistically significant differences between the wooden and metal materials groups regarding age (*p* > 0.999), sex (*p* = 0.702), weight (*p* = 0.834), classification Of PBI severity (*p* = 0.778), admission GCS score (*p* > 0.999), discharge GCS score (*p* > 0.999), depth of penetration (*p* = 0.746), and readmission (*p* = 0.063), follow up visit KPS score (*p* = 0.443). Late complications were observed in 12 patients, comprising cerebrospinal fluid leakage in 2 cases, headache in 1 case, epilepsy in 5 cases, and brain abscess in 4 cases, as detailed in Table 1.

**Table 1:** Comparison of clinical data between the two groups.

Variable	Over all *N* = 30^1^	Materials		Statistics	*p*-value^2^
		Metal *N* = 19 (63%)^1^	Wood *N* = 11 (37%)^1^		
Age(years)	8.00 (3.30)	8.00 (3.20)	8.00 (3.63)	0.00	>0.999
Sex					0.702
Male	20 (66.67%)	12 (63.16%)	8 (72.73%)		
Female	10 (33.33%)	7 (36.84%)	3 (27.27%)		
Weight(kg)	29.10 (10.40)	28.79 (9.86)	29.64 (11.75)	−0.21	0.834
Entry point					<0.001
Maxilar	1 (3.33%)	1 (5.26%)	0 (0.00%)		
Frontal	9 (30.00%)	9 (47.37%)	0 (0.00%)		
Orbit	9 (30.00%)	0 (0.00%)	9 (81.82%)		
Occiput	4 (13.33%)	4 (21.05%)	0 (0.00%)		
Mouth	2 (6.67%)	0 (0.00%)	2 (18.18%)		
Parietal	5 (16.67%)	5 (26.32%)	0 (0.00%)		
Classification of PBI severity					0.778
Mild	9 (30.00%)	6 (31.58%)	3 (27.27%)		
Moderate	15 (50.00%)	10 (52.63%)	5 (45.45%)		
Severe	6 (20.00%)	3 (15.79%)	3 (27.27%)		
Admission GCS score	10.50 [9.25, 13.00]	10.00[10.00, 13.00]	12.00 [8.50, 12.50]	104.00	>0.999
Discharge GCS score	15.00 [12.00, 15.00]	15.00 [12.00, 15.00]	15.00 [12.00, 15.00]	104.50	>0.999
Late complications					0.006
No	18 (60.00%)	14 (73.68%)	4 (36.36%)		
CSF leak	2 (6.67%)	2 (10.53%)	0 (0.00%)		
Headache	1 (3.33%)	1 (5.26%)	0 (0.00%)		
Epilepsy	5 (16.67%)	0 (0.00%)	5 (45.45%)		
Brain Abscess	4 (13.33%)	2 (10.53%)	2 (18.18%)		
Depth of penetration(cm)	2.10 [1.50, 3.43]	2.10 [1.65, 3.00]	2.10 [1.45, 3.65]	96.5	0.746
Readmission					0.063
Yes	12 (40.00%)	5 (26.32%)	7 (63.64%)		
No	18 (60.00%)	14 (73.68%)	4 (36.36%)		
Follow up visit KPS score	90.00 [72.50, 90.00]	90.00 [70.00, 90.00]	90.00 [80.00, 90.00]	88.00	0.443

1 Mean (SD); n (%); Median [IQR].

2 Two Sample *t*-test; Fisher’s exact test; Fisher’s Exact Test for Count Data with simulated *p*-value; Wilcoxon rank sum test. Follow up visit KPS score, **a**ll KPS scores were uniformly collected at the 3-month postoperative visit.

The 12 patients who experienced late complications are identical to those who were readmitted; all late complications necessitated inpatient management.

### Typical case

**Figure 1:**
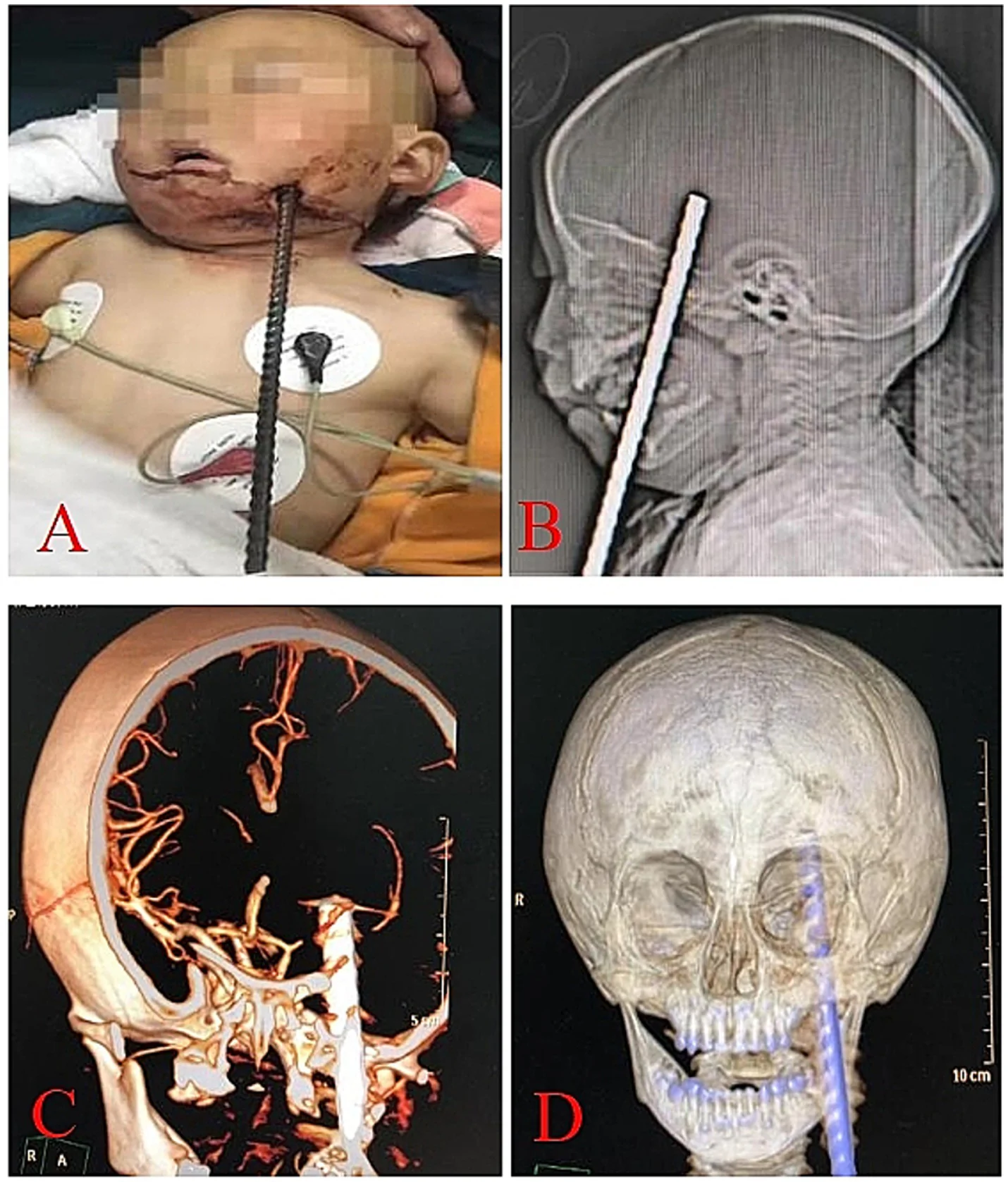
**(A)** Rebar penetrating the left upper jaw bone. **(B)** X-rays plain radiography demonstrating that the rebar penetrated about 5 cm into the skull. **(C)** Computed tomography angiography demonstrating left middle cerebral artery which was compressed by rebar. **(D)** Computed tomography reconstruction showing a hyperdense foreign material that penetrated from the left upper jaw bone to the deep structures in an oblique fashion.

Figure 1 illustrates pediatric patients with severe penetrating brain injuries. The penetrating object was a rebar that penetrated 5 cm into the skull and transferred from the upper jaw bone to the cranium. The surgical procedure was meant to achieve emergency treatment, repeated irrigation of the wound and operative area, and skull base reconstruction.

### Relevant factors for late complications

Upon comparing the baseline characteristics of the two patient groups, the analysis reveals no statistically significant differences in age, sex, weight, or materials between the groups (*p* > 0.05), as detailed in the accompanying table. Statistical analysis further indicates that sex (*p* = 0.694), weight (*p* = 0.748), and materials (*p* = 0.063) do not significantly influence the occurrence of late complications. Conversely, the variables ‘classification of PBI severity’ (*p* = 0.046) and ‘depth of penetration’ (*p* < 0.001) are identified as statistically significant determinants, as presented in Table 2.

**Table 2:** Analysis of relevant factors for late complications.

Variable	Overall N = 30^1^	Late complications		Statistics	*p*-value^2^
		No *N* = 18 (60%)^1^	Yes *N* = 12 (40%)^1^		
Age(years)	8.00 (3.30)	8.39 (2.97)	7.42 (3.80)	0.79	0.439
Sex					0.694
Male	20 (66.67%)	11 (61.11%)	9 (75.00%)		
Female	10 (33.33%)	7 (38.89%)	3 (25.00%)		
Weight(kg)	29.10 (10.40)	29.61 (9.31)	28.33 (12.25)	0.32	0.748
Materials					0.063
Metal	19 (63.33%)	14 (77.78%)	5 (41.67%)		
Wood	11 (36.67%)	4 (22.22%)	7 (58.33%)		
Classification of PBI severity					0.046
Mild	9 (30.00%)	7 (38.89%)	2 (16.67%)		
Moderate	15 (50.00%)	10 (55.56%)	5 (41.67%)		
Severe	6 (20.00%)	1 (5.56%)	5 (41.67%)		
Depth of penetration(cm)					<0.001
<3	20 (66.67%)	17 (94.44%)	3 (25.00%)		
>3	10 (33.33%)	1 (5.56%)	9 (75.00%)		

1 Mean (SD); n (%); Median [IQR].

2 Two Sample *t*-test; Fisher’s exact test; Fisher’s Exact Test for Count Data with simulated *p*-value; Wilcoxon rank sum test.

### Factors influencing the incidence of late complications

**Figure 2:**
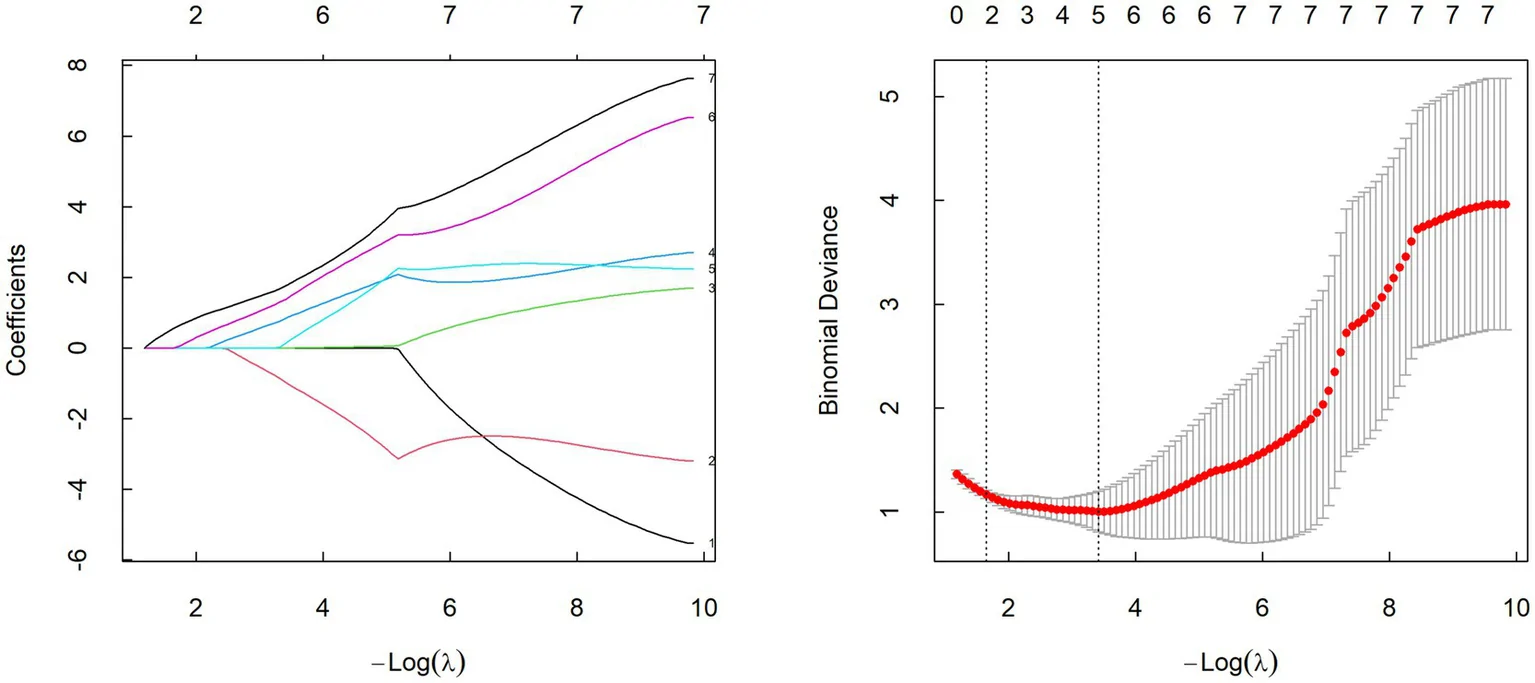
Selection of core predictors for long-term complications based on Lasso regression (left: Lasso regression coefficient path plot; right: Lasso regression cross-validation bias plot).

**Figure 3:**
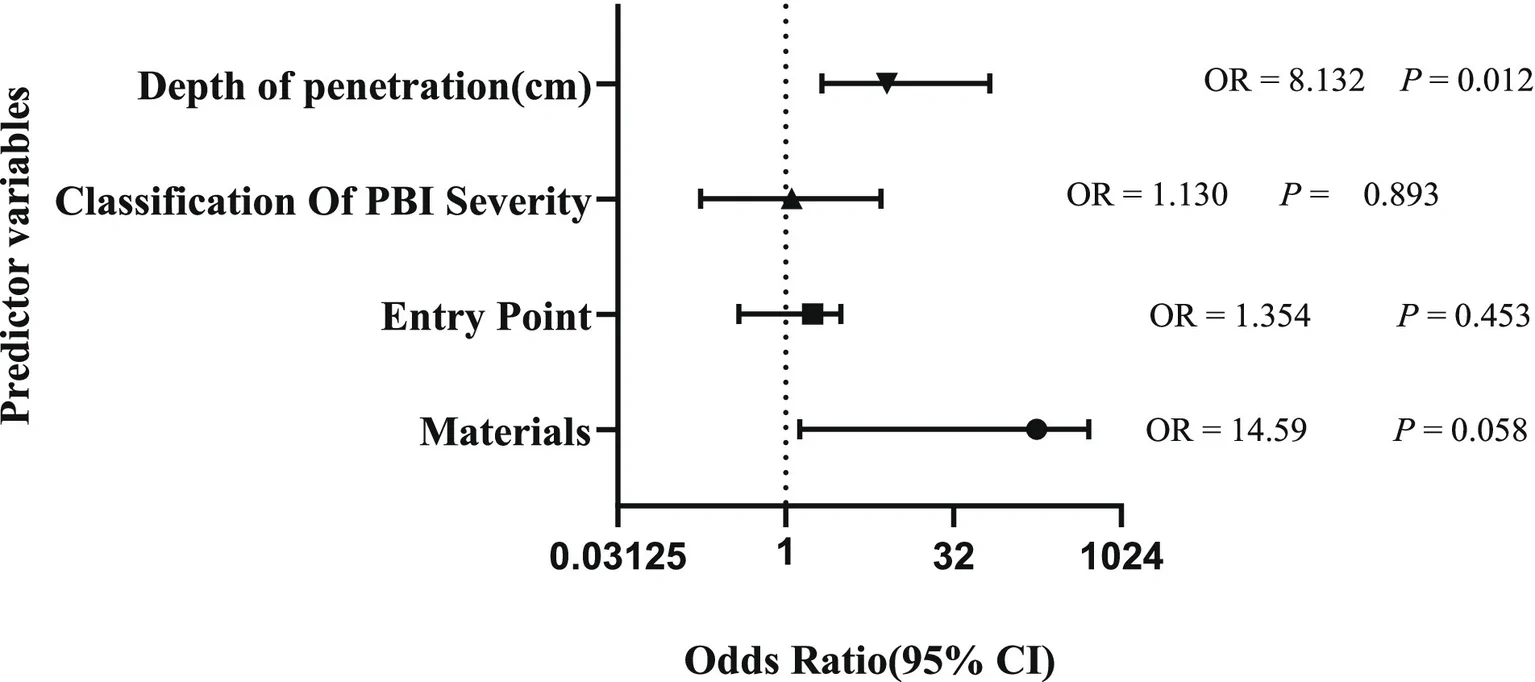
Forest plot showing odds ratio of multivariable logistic regression for the long-term complications in children. Risk variable indicated in brackets (materials; entry point; classification of PBI severity; depth of penetration).

Univariate and multivariate logistic regression were performed to explore risk factors for late complications (Table 3). In the univariate analysis, both the severity of brain-penetrating injury (OR 3.864, *p* = 0.038) and the depth of penetration (OR 5.867, *p* = 0.001) were identified as risk factors for delayed complications. To mitigate the risk of overfitting and to identify predictors with potential predictive value, we employed Lasso regression as an initial variable selection method (Table 4). After selection by the Lasso model, five key features with non-zero coefficients were ultimately determined to form the best prediction subset: penetration depth, classification of PBI severity, gender, material, and entry point. To quantitatively assess the reliability of the model, the area under the receiver operating characteristic curve (AUC) and the McFadden pseudo-R2 were computed. The findings indicated that the multivariate model exhibited exceptional discrimination and statistical reliability, with an AUC value of 0.9676 and a McFadden pseudo-R^2^ of 0.6872 (Figure 2). It is widely recognized that gender does not influence long-term prognosis. Consequently, this variable was excluded from our multivariate analysis. However, in the multivariate analysis, depth of penetration (OR 8.132, *p* = 0.012) was established as an independent risk factor for the occurrence of delayed complications. Forest plot (Figure 3) were utilized to analyze the association between the late complications and additional contributing factors materials, entry point, classification of PBI severity, depth of penetration.

**Table 3:** Analysis of risk factors influencing the late complications in children.

Variable	Crude	Adjusted
	OR (95% CI) *p* value	OR (95% CI) *p* value
Weight(kg)	0.988 (0.916 to 1.062) 0.738	
Age(years)	0.910(0.711 to 1.142) 0.425	
Sex	0.524(0.092 to 2.508) 0.432	
Materials	0.275 (0.054 to 1.241) 0.101	14.59 (1.336 to 521.3) 0.058
Entry point	0.950 (0.562 to 1.563) 0.840	1.354 (0.622 to 3.269) 0.453
Classification of PBI severity	3.864 (1.208 to 17.12) 0.038	1.130 (0.173 to 7.199) 0.893
Depth of penetration(cm)	5.867 (2.159 to 23.52) 0.001	8.132 (2.116 to 67.90) 0.012

**Table 4:** Lasso regression feature selection results.

Variable name	Lasso coefficient
Depth of penetration(cm)	1.7709
Classification of PBI severity	1.3814
Sex	−0.9816
Materials	0.8405
Entry point	0.1400
Age(years)	0
Weight(kg)	0

### Relevant factors for late epilepsy

A comparative analysis of the baseline characteristics between the two patient groups reveals no statistically significant differences in sex, weight, depth of penetration, classification of PBI severity (*p* > 0.05), as illustrated in the accompanying table. Statistical evaluations of these factors indicate that sex (*p* > 0.999), weight (*p* = 0.319), classification of PBI severity (*p* = 0.051), and depth of penetration (*p* > 0.999) do not serve as significant determinants of late-onset epilepsy. Conversely, the variables of materials (*p* = 0.003) and entry point (*p* = 0.018) are identified as statistically significant factors, as detailed in Table 5.

**Table 5:** Analysis of relevant factors for late epilepsy.

Variable	Overall, *N* = 30^1^	Late epilepsy		Statistics	*p*-value^2^
		No *N* = 25 (83%)^1^	Yes *N* = 5 (17%)^1^		
Sex					>0.999
Male	20 (66.67%)	17 (68.00%)	3 (60.00%)		
Female	10 (33.33%)	8 (32.00%)	2 (40.00%)		
Weight (kg)	29.10 (10.40)	29.96 (10.66)	24.80 (8.64)	1.01	0.319
Materials					0.003
Metal	19 (63.33%)	19 (76.00%)	0 (0.00%)		
Wood	11 (36.67%)	6 (24.00%)	5 (100.00%)		
Depth of penetration(cm)					>0.999
<3	20 (66.67%)	17 (65.38%)	3 (75.00%)		
>3	10 (33.33%)	9 (34.62%)	1 (25.00%)		
Classification of PBI severity					0.051
Mild	9 (30.00%)	9 (36.00%)	0 (0.00%)		
Moderate	15 (50.00%)	13 (52.00%)	2 (40.00%)		
Severe	6 (20.00%)	3 (12.00%)	3 (60.00%)		
Entry point					0.018
Maxilar	1 (3.33%)	1 (4.00%)	0 (0.00%)		
Frontal	9 (30.00%)	9 (36.00%)	0 (0.00%)		
Parietal	5 (16.67%)	5 (20.00%)	0 (0.00%)		
Occiput	4 (13.33%)	4 (16.00%)	0 (0.00%)		
Orbit	9 (30.00%)	4 (16.00%)	5 (100.00%)		
Mouth	2 (6.67%)	2 (8.00%)	0 (0.00%)		

1 n (%); Mean (SD).

2 Fisher’s exact test; Two Sample t-test; Fisher’s Exact Test for Count Data with simulated *p*-value.

## Discussion

Craniocerebral injuries are becoming very common in children, which is highly attributed to hyperactivity, with falls being the main cause of head injuries in children. However, traffic accident trauma causes more severe injuries. In children and adolescents, it is necessary to obtain a detailed case history that will clarify the injury type, the height from which the child had fallen, the floor characteristics (carpeted or not), the initial state of consciousness (crying or not), and the onset and occurrence of apnea (1).

The consciousness stage is quantified using the GCS grading system (6). The GCS is a useful tool that should be used carefully and precisely. A few studies reported a good correlation between GCS stage and neurologic outcome (7–9). Occasionally, penetrating wounds are reported. Currently, accidental brain injury is the most common PBI (10). The direction of the penetrating foreign body determines its trajectory and extent of injury (11). Rebar can directly penetrate objects posterior to vital structures such as the optic nerve, cavernous sinus, brainstem, and temporal lobe. However, a foreign body penetrating the orbit, the lower jawbone, or the nose can penetrate the sinuses and intracranial cavity (12). These pathways occur through the thin orbital roof, which may damage the optic nerve and superior orbital (13, 14). Furthermore, foreign bodies can be directed up the narrow nasal cavity to the cribriform plate and penetrate the intracranial cavity (11, 12, 15–20).

### The penetrating object

Different types of foreign bodies and penetrating injuries in different parts of the body have specific characteristics: clinical symptoms and CT scans of wooden foreign bodies entering the skull might not be characteristic, only demonstrating low-density shadows, which might result in missed or delayed diagnosis (21). Hiraishi et al. (22) suggested that the possibility of penetrating injury-causing intracranial wooden foreign bodies should be considered in unexplained intracranial abscesses in children. Metal foreign bodies have harder textures than the skull and thus often cause comminuted fractures of the skull and skull base, which may result in severe brain contusions or intracranial hematoma.

### Preoperative neuroradiologic assessment

After penetrating cranial trauma, the patient must be referred for a detailed neuroradiologic evaluation to verify the object trajectory and occurrence of secondary lesions to guide the best neurosurgical management. Children’s nervous systems are not fully developed, and their skulls are thin. Therefore, individualized treatment plans should be designed according to the characteristics of the foreign body that caused the craniocerebral puncture and wound. Head CT and three-dimensional reconstruction should be performed immediately to elucidate intracranial injuries among children with PBIs. Furthermore, brain CTA should be performed as soon as possible to determine whether the object has penetrated intracranial vessels. Importantly, these tests are used to prepare for surgery. CT is important in the early diagnosis of craniocerebral injuries. Cerebral angiography should be performed to identify branching vessel injuries when the injury trajectory affects bleeding sites such as the internal jugular and intracranial arteries, lateral fissures, cavernous sinus, and great venous sinus (23–25). Digital subtraction angiography (DSA) is the gold standard for establishing the relationship between the penetrating substance and intracranial vessels (26). Nevertheless, CTA is often used instead of DSA to observe blood vessels and blood flow in emergency or limited conditions. CT three-dimensional reconstruction is very useful for establishing the relationship between foreign bodies and the skull base and skull structures. However, CTA is also preferred as it is easy to perform (27).

### Neurosurgical management

Perioperative management is aimed at preventing infections, epilepsy, and cranial hypertension. Skull base penetrating injuries due to foreign body residues result in severe and complex open injuries. The PBI treatment objectives are to (i) remove the intracranial foreign body, (ii) perform debridement to prevent sinus tract and operative area infections, (iii) confirm whether there is vascular injury, and (iv) prevent CSF leakage.

Children’s skulls are thin, soft, elastic, and have poor resistance to violent impacts. Their nervous system development is immature, and their neural function has strong plasticity (28). Therefore, personalized treatment plans for children with intracranial and extracranial penetrating injuries should be selected based on the characteristics and different parts of the foreign body.

### Late complications associated with penetrating brain injuries

Penetrating brain injuries in children can result in profound damage to the central nervous system, often leading to high rates of mortality and disability. Those who survive may experience enduring complications that substantially impact their quality of life. This study seeks to investigate the risk factors contributing to long-term complications in pediatric patients, with a specific emphasis on the prevalence of such complications in children who have sustained penetrating brain injuries and the associated risk factors. The most frequent etiologies causing late-onset epilepsy (LOE) are cerebrovascular disorders (CVDs) and stroke (29).

Prior studies on intracranial foreign bodies and delayed brain abscesses highlight that the physical properties and remnants of foreign materials significantly contribute to long-term infection risks after cranial reconstruction and head trauma. Wooden and bamboo materials, including cranial implants, are porous and brittle, often leaving microscopic debris in the brain that fosters bacterial biofilm growth. One case showed that wooden brain injuries could lead to delayed cerebellar abscesses despite proper antibiotic use, indicating antibiotics alone cannot eliminate infection risks from organic residues. Another pediatric case of bamboo stick injury revealed that small skin wounds can conceal deep intracranial plant-based foreign bodies, initially showing minimal symptoms but later developing a mature frontal abscess (22, 30, 31).

Nevertheless, several limitations should be acknowledged. This study was conducted at a single tertiary pediatric neurosurgical center with a limited sample size, which restricts the generalizability of our conclusions. At present, there is insufficient evidence to suggest that wooden materials are more likely to cause brain abscesses compared to metal materials. The potential difference remains to be further verified through rigorous clinical and experimental studies.

Based on the risk factors identified in our study, we categorize patients into specific high-risk subgroups and propose corresponding clinical screening and intervention strategies, as detailed below. Enhanced surveillance protocols are implemented for high-risk patients, with a focus on neuroimaging and clinical assessment. Specifically, contrast-enhanced cranial MRI is conducted at 1, 3, 6, 12, and 24 months postoperatively, in lieu of the traditional non-contrast CT. This preference for MRI is due to its superior ability to detect early micro-abscesses, granulation tissue, and small residual implant fragments, which are often overlooked by CT bone window scanning. In terms of clinical assessment, a standardized approach is employed, emphasizing the identification of subtle prodromal infectious symptoms such as intermittent headache, low-grade fever, hyposmia, and lethargy, alongside routine neurological examinations at each follow-up appointment. Additionally, caregivers are instructed to seek immediate medical attention should any concerning symptoms arise.

### Study limitations

This single-center retrospective study has limited generalizability. Using data from multiple centers and surgical teams would provide stronger evidence. Additionally, the small sample size and few clinical outcomes introduce statistical bias. Due to the relatively small sample size and the exploratory nature of this study, the results of the multivariable logistic regression should be interpreted with caution. A larger independent cohort is required to validate the observed associations.

## Conclusion

PBIs are rare occurrences, predominantly arising from accidents and interpersonal violence. Acknowledging the intrinsic limitations of this retrospective, single-institution cohort study, we present a synthesis of our institutional findings concerning late complications following PBIs in pediatric patients. Our observations indicate that the depth of penetration may serve as a potential risk factor for subsequent late adverse events. Furthermore, statistical analyses suggest that the material of the injury and entry point are associated with the late epilepsy. Due to the small sample size and limited control of confounding factors, this association is exploratory and needs confirmation through larger prospective or multicenter studies.

## Data Availability

The original contributions presented in the study are included in the article/supplementary material, further inquiries can be directed to the corresponding authors.
